# Draft Genome Sequence of *Shewanella* sp. Strain T2.3D-1.1, Isolated from 121.8 Meters Deep in the Subsurface of the Iberian Pyrite Belt

**DOI:** 10.1128/MRA.00190-20

**Published:** 2020-10-01

**Authors:** Sofia de Francisco-Polanco, José M. Martínez, Tania Leandro, Ricardo Amils

**Affiliations:** a Centro de Biología Molecular Severo Ochoa (CBMSO, CSIC-UAM), Universidad Autónoma de Madrid, Madrid, Spain; b Institute for Bioengineering and Biosciences (IBB), Instituto Superior Técnico, Lisbon, Portugal; c Centro de Astrobiología (CAB, CSIC-INTA), Madrid, Spain; University of Southern California

## Abstract

*Shewanella* sp. strain T2.3D-1.1 was isolated from the deep subsurface of the Iberian Pyrite Belt. We report its draft genome sequence, consisting of 49 scaffolds, with a chromosome of ≈4.6 Mb and a 23.8-kb plasmid. The chromosome annotation identified 4,068 coding DNA sequences, 1 rRNA operon, and 108 tRNA genes.

## ANNOUNCEMENT

*Shewanella* is the only genus in the family *Shewanellaceae* within the order *Alteromonadales*. Species of this genus are Gram-negative, motile rods which are widely distributed in many environments. Their growth is facultatively anaerobic, and they can use a wide range of compounds as electron acceptors (1). *Shewanella* sp. strain T2.3D-1.1 was isolated from a strictly anaerobic denitrification enrichment culture using a 121.8-m-deep core sample obtained from a drilling project aimed at studying the microbial diversity existing in the deep subsurface of the Iberian Pyrite Belt (southwest Spain) (2). The borehole coordinates were 37°43′45.42″N and 6°33′23.57″W. Drilling was performed as described in reference 3. A powdered rock sample (≈6 g) was used as the inoculum. *Shewanella* sp. strain T2.3D-1.1 was isolated and grown under the conditions described in reference 4.

Genomic DNA of the strain T2.3D-1.1 was extracted by the cetyltrimethylammonium bromide (CTAB)-based method (5), and its concentration was determined using a Qubit v.2.0 fluorometer (Invitrogen, USA). The 16S rRNA gene was amplified under the conditions described in reference 4. Reads were edited and assembled as described in reference 6. The complete 16S rRNA gene sequence was compared with sequences in the NCBI GenBank database using BLAST (7). The closest sequence was found to belong to Shewanella hafniensis P010(T) (99.43% similarity) (8).

Library preparation and sequencing were carried out by the MicrobesNG company using the Illumina MiSeq platform, with a mean coverage of 73.37×. The sequencing run generated 763,338 paired-end reads of 2 × 250 bp with a mean length of 537 bp. *De novo* assembly was carried out using SPAdes v.3.14.0 (“-careful” option) (9), and the extrachromosomal elements were assembled with Recycler, which uses the read coverage of contigs to distinguish between plasmids and chromosomes (10). Plasmid contigs were aligned against the chromosomal assembly with Mauve Aligner v.2.4.0 (11) to separate chromosomal and plasmid contigs. Contigs were extended and merged into scaffolds using SSPACE software (12). Gaps created with SSPACE were closed with GapFiller v.1-10 software (13). Default parameters were used for all software unless otherwise specified. This yielded a chromosome in 49 scaffolds, with an *N*_50_ value of 183,411 bp, a GC content of 44.42%, 1 chromosome of 4,660,397 bp, and 1 plasmid of 23,806 bp in 3 scaffolds.

The genome was annotated with Prokka v.1.12 software (14) and the RAST platform (15), using Shewanella putrefaciens strain 200 as the reference genome. A total of 4,068 coding DNA sequences, 108 tRNA genes, 1 rRNA operon, 1 transfer-messenger RNA (tmRNA), and 3 CRISPR sequences were identified. Genes involved in (i) denitrification, (ii) both assimilatory and dissimilatory sulfur reduction pathways, (iii) different reductase enzymes which are part of anaerobic respiration, (iv) a ferric ion reductase, and (v) heavy metal resistance were identified in the chromosome.

The analysis of the genome of *Shewanella* sp. strain T2.3D-1.1 should assist in identifying the mechanisms used by organisms to develop under the extreme oligotrophic conditions present in the dark biosphere.

### Data availability.

Reads were deposited at ENA/GenBank/DDBJ under the accession number ERR3773752, and the complete genome sequences were deposited under the accession numbers CACVBT020000000 (chromosome) and CACVAL020000000 (plasmid). All of them are included under study number PRJEB35936.

## References

[1] Nealson KH, Scott J 2006 Ecophysiology of the genus Shewanella, p 1133–1151. *In* Dworkin M (ed), The prokaryotes. Springer, New York, NY.

[2] Amils R, Fernández-Remolar D, Parro V, Rodríguez-Manfredi JA, Timmis K, Oggerin M, Sánchez-Román M, López JF, Fernández JP, Puente F, Gómez-Ortiz D, Briones C, Gómez F, Omoregie E, García M, Rodríguez N, Sanz JL 2013 Iberian Pyrite Belt subsurface life (IPBSL), a drilling project of biohydrometallurgical interest. Adv Mater Res 825:15–18. doi:10.4028/www.scientific.net/AMR.825.15.

[3] Puente-Sánchez F, Arce-Rodríguez A, Oggerin M, García-Villadangos M, Moreno-Paz M, Blanco Y, Rodríguez N, Bird L, Lincoln SA, Tornos F, Prieto-Ballesteros O, Freeman KH, Pieper DH, Timmis KN, Amils R, Parro V 2018 Viable cyanobacteria in the deep continental subsurface. Proc Natl Acad Sci U S A 115:10702–10707. doi:10.1073/pnas.1808176115. 30275328 PMC6196553

[4] García R, Martínez JM, Leandro T, Amils R 2018 Draft genome sequence of Rhizobium sp. strain T2.30D-1.1, isolated from 538.5 meters deep on the subsurface of the Iberian Pyrite Belt. Microbiol Resour Announc 7:e01098-18. doi:10.1128/MRA.01098-18. 30533741 PMC6256577

[5] Wilson K 2001 Preparation of genomic DNA from bacteria. Curr Protoc Mol Biol 56:2.4.1–2.4.5. doi:10.1002/0471142727.mb0204s56. 18265184

[6] Mariñán N, Martínez JM, Leandro T, Amils R 2019 Draft genome sequence of Rhodoplanes sp. strain T2.26MG-98, isolated from 492.6 meters deep on the subsurface of the Iberian Pyrite Belt. Microbiol Resour Announc 8:e00070-19. doi:10.1128/MRA.00070-19. 31000538 PMC6473132

[7] Benson DA, Karsch-Mizrachi I, Lipman DJ, Ostell J, Wheeler DL 2008 GenBank. Nucleic Acids Res 36:25–30. doi:10.1093/nar/gks1195. PMC2238942 18073190

[8] Satomi M, Vogel BF, Gram L, Venkateswaran K 2006 Shewanella hafniensis sp. nov. and Shewanella morhuae sp. nov., isolated from marine fish of the Baltic Sea. Int J Syst Evol Microbiol 56:243–249. doi:10.1099/ijs.0.63931-0. 16403893

[9] Bankevich A, Nurk S, Antipov D, Gurevich AA, Dvorkin M, Kulikov AS, Lesin VM, Nikolenko SI, Pham S, Prjibelski AD, Pyshkin AV, Sirotkin AV, Vyahhi N, Tesler G, Alekseyev MA, Pevzner PA 2012 SPAdes: a new genome assembly algorithm and its applications to single-cell sequencing. J Comput Biol 19:455–477. doi:10.1089/cmb.2012.0021. 22506599 PMC3342519

[10] Rozov R, Kav AB, Bogumil D, Shterzer N, Halperin E, Mizrahi I, Shamir R 2017 Recycler: an algorithm for detecting plasmids from de novo assembly graphs. Bioinformatics 33:475–482. doi:10.1093/bioinformatics/btw651. 28003256 PMC5408804

[11] Darling ACE, Mau B, Blattner FR, Perna NT 2004 Mauve: multiple alignment of conserved genomic sequence with rearrangements. Genome Res 14:1394–1403. doi:10.1101/gr.2289704. 15231754 PMC442156

[12] Boetzer M, Henkel CV, Jansen HJ, Butler D, Pirovano W 2011 Scaffolding pre-assembled contigs using SSPACE. Bioinformatics 27:578–579. doi:10.1093/bioinformatics/btq683. 21149342

[13] Nadalin F, Vezzi F, Policriti A 2012 GapFiller: a de novo assembly approach to fill the gap within paired reads. BMC Bioinformatics 13:S8. doi:10.1186/1471-2105-13-S14-S8. PMC3439727 23095524

[14] Seemann T 2014 Prokka: rapid prokaryotic genome annotation. Bioinformatics 30:2068–2069. doi:10.1093/bioinformatics/btu153. 24642063

[15] Aziz RK, Bartels D, Best A, DeJongh M, Disz T, Edwards RA, Formsma K, Gerdes S, Glass EM, Kubal M, Meyer F, Olsen GJ, Olson R, Osterman AL, Overbeek RA, McNeil LK, Paarmann D, Paczian T, Parrello B, Pusch GD, Reich C, Stevens R, Vassieva O, Vonstein V, Wilke A, Zagnitko O 2008 The RAST server: Rapid Annotations using Subsystems Technology. BMC Genomics 9:75. doi:10.1186/1471-2164-9-75. 18261238 PMC2265698

